# Theoretical study on the influence of different exciton Hamiltonians on the excitation dynamic process of the PE555 complex

**DOI:** 10.1039/d5ra09996j

**Published:** 2026-05-07

**Authors:** XueYan Cui, ZeMin Sheng, Wei Song, Dengke Zhao, YiJing Yan, JianHua Wei

**Affiliations:** a Department of Science, Henan Institute of Technology Xinxiang 453003 China cxy@hait.edu.cn; b School of Materials Science and Engineering, Henan Normal University Xinxiang 453007 China; c Department of Chemical Physics, Hefei National Laboratory for Physical Sciences at the Microscale, University of Science and Technology of China Hefei Anhui 230026 China; d Department of Physics, Beijing Key Laboratory of Optoelectronic Functional Materials and Micro-nano Devices, Renmin University of China Beijing 100872 China

## Abstract

Photosynthesis initiates at the light harvesting stage, where specific pigment–protein complexes can convert absorbed light energy into electronic excited states. These excited states are transferred to the reaction center, initiating charge separation. Existing studies have confirmed that quantum coherence between electronic excited states plays a pivotal role in excitation energy transfer. In this paper, we employ the dissipation equation of motion (DEOM) method from quantum dissipation theory to investigate the influence of the structure of the PE555 complex on its exciton dynamics, while also exploring the quantum coherence characteristics during the excitation energy transfer process of the complex. The research focuses on examining the effects of exciton-type Hamiltonians obtained by two different methods (the point dipole approximation (PDA) method and the transition charge from electrostatic potential (TrEsp) method) on the aforementioned processes. The results demonstrate that, under both low temperature and room temperature conditions, when the Hamiltonian obtained by the PDA method is combined with the DEOM method for calculations, the excitation energy transfer in the PE555 complex is faster, and more energy is transferred to the DBV50/61B molecule. The analysis reveals that the open structure of the PE555 complex results in larger exciton coupling values obtained by the PDA method, with a wider coupling distribution compared to that from the TrEsp method, which directly facilitates the efficient transfer of excitation energy. This study provides a theoretical basis for understanding the regulatory mechanisms of quantum coherence effects and structural characteristics on energy transfer in light harvesting systems.

## Abstract 2

Photosynthesis initiates at the light harvesting stage, where specific pigment–protein complexes can convert absorbed light energy into electronic excited states.

## Graphical abstract

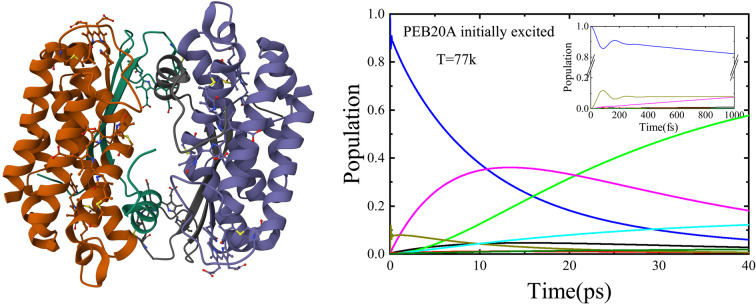

## Introduction

Life on Earth has utilized solar energy through the mechanism of photosynthesis for 4 billion years. Through evolution, photosynthetic antenna complexes have emerged to efficiently capture solar energy and transfer it to reaction centers, where the energy is stored as biochemical energy.^1^ The biological significance of photosynthesis is undeniable, as it forms the foundation for nearly all life on Earth. The initial step of photosynthesis involves capturing sunlight, for which specialized pigment–protein complexes, known as light harvesting antenna complexes, have evolved. The light harvesting complex is composed of chromophores. This process begins with the absorption of light energy by chromophores, exciting molecules from their ground state to an electronic excited state. Light harvesting complexes transfer the absorbed energy through a series of electronic energy transfer (EET) steps, ultimately reaching reaction centers with high quantum efficiency, despite significant diversity in antenna complex size, structure, and pigment composition. The ability of photosynthesis to transfer energy efficiently is crucial to its success, achieving efficiencies of nearly 99% in some bacterial systems.^2^ This remarkable efficiency has attracted extensive research across disciplines such as bioengineering, physics, and materials science, providing critical principles for designing biomimetic solar cells. Harnessing such high quantum efficiency enables the efficient conversion of solar energy, supporting our growing need for renewable green energy.^3–5^

The quantum coherence discussed in this work refers to the quantum superposition formed between the electronic excited states of different chromophores within the light-harvesting complex PE555. Fundamentally, it is manifested as a well-defined and temporally persistent phase relationship of the excited-state wavefunction in both spatial and temporal domains. This phase correlation constitutes the essential quantum-mechanical mechanism enabling non-classical energy transfer among chromophores. During the excitation energy transfer (EET) process in the PE555 complex, quantum coherence is specifically reflected in the quantum correlations between electronic excited states. When significant excitonic coupling exists among chromophores, excitation energy is no longer transferred in a classical hopping manner between localized excited states of individual chromophores. Instead, coherent interactions give rise to delocalized excitonic states, in which the excitation energy is distributed over multiple chromophores in a superposition state. The sustained maintenance of phase relationships leads to population oscillations, namely periodic temporal variations in the excited-state populations of different chromophores. Quantum beating refers precisely to the oscillatory signatures of such population oscillations observed in spectroscopic signals or dynamical traces, and is therefore regarded as a direct experimental and theoretical manifestation of quantum coherence. In 2007, ultrafast two-dimensional electronic spectroscopy(2DES) was used to observe long-lived quantum beats in the Fenna-Matthews-Olson (FMO) complex at 77 K, with a duration exceeding 600 femtoseconds.^6^ In this case, the quantum beats were considered to be electronic quantum coherences because their oscillation frequencies correspond to the energy differences between excitons. The persistence of these quantum coherences has an impact on the energy transfer process in photosynthesis. These results triggered extensive research on quantum coherence, and subsequently, coherences were observed at both low temperatures and room temperatures.^7–9^ Other studies have shown that quantum coherence can be observed in various photosynthetic systems, including light harvesting protein–pigment complexes such as LH1 (ref. 10) and LH2,^11–13^ as well as in reaction centers.^14–17^ Recently, long-lived quantum coherences have been experimentally observed in the phycoerythrin 545 (PE545) antenna of the marine cryptophyte Rhodomonas sp. CS24.^8,18–20^ Currently, two-dimensional electronic spectroscopy of the cryptophyte complex PE555 have also been experimentally obtained.^21,22^ While these experimental observations have generated significant enthusiasm regarding the existence of quantum biological effects at room temperature, the field is consistently accompanied by profound critical discussions and cautious interpretations. Recent research^23^ points out that in complex biological environments, precisely distinguishing between electronic coherence, vibrational coherence, and mixed vibronic (vibrational-electronic) coherence presents a significant challenge; the physical origin of the observed quantum beat signals remains contentious. Furthermore, although theoretical models have demonstrated how coherence could potentially optimize energy transfer pathways, a core unresolved question persists: in the noisy room-temperature environment, is this seemingly fragile quantum effect truly exploited by evolution and does it constitute the cause of the nearly perfect energy transfer efficiency? or is it merely an epiphenomenon arising from strong molecular couplings and specific protein scaffolding, with the high efficiency of energy transfer primarily governed by the static energy funnel designed by the protein architecture? these open questions necessitate a critical approach when interpreting both theoretical and experimental results. The aim of our study is not simply to assert the presence or absence of quantum coherence. Rather, it is to employ precise quantum dynamical simulations to quantify, within a specific biological system (the PE555 complex), how different structural features influence the lifetime and extent of coherent dynamics *via* induced excitonic couplings, and how these in turn affect the final transfer efficiency. Thereby, our work aims to provide quantitative, mechanistic insights into the aforementioned debates. This paper aims to discuss the ultrafast energy transfer processes in PE555 complex, particularly the role of quantum coherences in energy transfer process, a topic that has attracted significant attention in recent experimental and theoretical research.

**Fig. 1:**
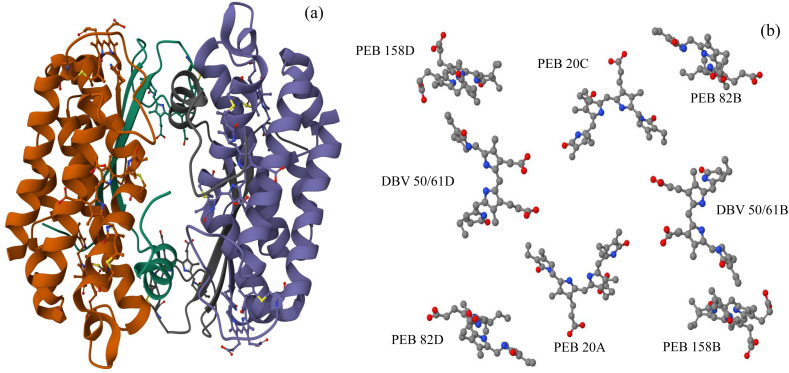
(a) the PE555 complex features a protein scaffold within which eight chromophore molecules are embedded. (b) The eight chromophore molecules form the structural basis of the PE555 complex.

Cryptophytes are eukaryotic algae that inhabit marine and freshwater environments. They are important primary producers, capable of capturing sunlight through adjustable linear tetrapyrrole chromophores called bilins. Despite their seemingly simple light harvesting antennas compared to other algae, cryptophytes exhibit a variety of colors and show maximum photosynthetic activity under extremely low light conditions.^24^ Based on their maximum absorption wavelengths, they are classified into two categories: phycoerythrins (PE) and phycocyanins (PC). To date, four types of PE (PE545, PE555, and two types of PE566) and five types of PC (PC569, PC577, PC612, PC630, and PC645) have been reported.^24–26^ The PE555 complex is a light harvesting system derived from the marine alga Hermiselmis andersenii CCMP 644, and its crystal structure has been resolved, with the PE555 from Hermiselmis andersenii determined at a resolution of 1.8 Å.^21^ This study focuses primarily on the phycoerythrin PE555. The protein structure of the PE555 complex is shown in Fig. 1. The PE555 complex contains six phycoerythrobilin (PEB) chromophore molecules and two dihydrobiliverdin (DBV) chromophore molecules, with its structure composed of four subunits (A, B, C, and D) forming a dimer of two *αβ* monomers. Among them, the *β* subunit contains two PEBs and one DBV, specifically *β*82 (PEB82B, 82D), *β*158 (PEB158B, 158D), and *β*50/*β*61 (DBV50/61B, 50/61D); the first PEB in the *α* subunit is labeled as *α*20 (PEB20A, 20C). Interestingly, over the past decade, a new “open” quaternary structure of cryptophyte antennas has been discovered in various species of the Hermiselmis lineage. This structure involves the insertion of a single aspartic acid residue in the α subunit sequence, causing the two αβ protomers to rotate by approximately 73°. This rotation leads to a significant reduction in electronic interactions between the central bilin pigments. This structural change is also associated with a decrease in quantum beats observed in two-dimensional electronic spectroscopy, which may be related to the involvement of vibrational coherence in the excitation energy transfer (EET) mechanism.^21^

Theoretical models describing the excitation energy transfer process in light harvesting systems have continuously evolved through in-depth research, encompassing multiple dimensions, from classical to quantum and from static to dynamic. The applicability of the classical Förster theory^27–29^ in the excitation energy transfer of light harvesting systems requires analysis based on the specific characteristics of the system. The core assumption of this theory is that excitation energy is transferred in the form of incoherent “hopping” between localized chromophore states, and its validity relies on two key premises: first, the electronic coupling strength between chromophores is much weaker than the energy disorder caused by environmental fluctuations; second, electronic coherence (the quantum superposition of excited states in different chromophores) decays rapidly due to environmental disturbances (such as protein vibrations and solvent fluctuations) and thus cannot participate in energy transfer.^30^ These conditions can be approximately satisfied in some simple light harvesting systems with loose structures and large chromophore distances (*e.g.*, isolated antenna proteins of certain lower algae). However, the limitations of Förster theory become significantly prominent in most efficient light harvesting complexes (*e.g.*, LH2 in higher plants, phycobilisomes in cyanobacteria, or PE555 in cryptophytes). In these systems, chromophores are closely packed to enhance the light absorption cross-section, resulting in a significant increase in electronic coupling strength and the formation of stable “exciton states” (*i.e.*, excitation energy is delocalized among multiple chromophores), which directly violates the theory's assumption of “localized single-chromophore states”. Meanwhile, experiments using two-dimensional electronic spectroscopy have observed long-lived quantum coherence (such as coherent oscillations exceeding 600 femtoseconds in the FMO complex),^6^ indicating that environmental disturbances do not destroy electronic coherence, and energy transfer may occur through the quantum superposition mechanism of coherent states rather than mere incoherent hopping.

It is noteworthy that the excitonic coupling strengths in this work are calculated using the point dipole approximation (PDA). This approach neglects contributions from higher-order multipole moments as well as short-range exchange interactions. As discussed by Fleming, Scholes and co-workers,^31^ the PDA may introduce quantitative deviations when the inter-pigment distance is small or when there is significant spatial overlap of the transition charge densities. However, for the typical pigment separations in the PE555 complex, the dipole–dipole interaction remains a reasonable first-order approximation to the coulombic coupling. Although more sophisticated approaches (such as the transition density cube method or *ab initio* electrostatic potential calculations) could further improve the quantitative accuracy of the coupling strengths, the qualitative features of the energy transfer pathways and coherent dynamics discussed here are not expected to be substantially altered. Therefore, in complex systems where chromophores are closely arranged with significant electronic coupling and quantum coherence, more comprehensive frameworks such as exciton theory and non-Markovian quantum dynamics must be combined to accurately describe the excitation energy transfer mechanism.^32^

Recent studies^33,34^ have claimed that quantum coherence is crucial for achieving efficient light harvesting, and accumulating evidence indicates that coherent effects are involved in the dynamics and mechanisms of ultrafast light harvesting. The key to efficiency lies in the balance between the ultrafast (femtosecond to picosecond) transfer time of electronic excitation between antenna complexes, the relaxation time to the ground state, and the relationship between excitation diffusion time and antenna size.^35,36^ Therefore, many biophysical studies have focused on elucidating the mechanism of such energy transfer. In particular, extensive theoretical and experimental studies have been conducted on the FMO complex;^37–40^ however, research on cryptophyte proteins remains relatively scarce. This paper primarily investigates the exciton dynamics in the PE555 complex, explores the role of quantum coherence in the excitation energy transfer process, and further studies its exciton dynamics.

In this article, through quantum simulations, we mainly discuss whether the pigment–protein coupling properties in the chromophore network play an important role in the excitation energy transfer (EET) in the PE555 complex. Using the system Hamiltonian obtained by two different electronic coupling methods, we investigate the exciton dynamics of the PE555 complex by adopting the dissipation equation of motion (DEOM) method. In addition, we explore that the interaction between the system and the solvent protein environment significantly affects the excitation dynamics. The calculation results show that there exist long-lived quantum coherent oscillations during the population transfer in the PE555 complex. For the PE555 complex, the calculation results indicate that the coupling between pigments and the protein environment plays an important role in the excitation energy transfer process.

## Model and theory

Research on exciton dynamics in the PE555 complex, the relatively large average distance between chromophore molecules causes the excitation energy to exhibit delocalized characteristics, and this phenomenon can be explained within the theoretical framework of the Frenkel exciton model. We employed an exciton model constructed upon a full Hamiltonian derived from the structural calculations of the PE555 complex. Electronic coupling is defined as the coulomb interaction between the electronic transition densities (or transition dipole moments) of chromophores *m* and *n*. It corresponds to the off-diagonal elements in the Frenkel exciton Hamiltonian and quantitatively characterizes the strength of excitation energy transfer between chromophores. Electronic coupling is used synonymously with electronic coupling within the framework of the Frenkel exciton model. For chromophore–protein interactions and intermolecular interactions between chromophores that do not form excitonic states, we uniformly use the term coupling. The calculation of electronic coupling in the PE555 complex is based on previous studies.^21,41^ Chandrasekaran *et al.*^41^ performed ground state molecular dynamics (MD) simulations using a non-polarizable force field and calculated the excited states along this trajectory by employing Zerner's Intermediate Neglect of Differential Overlap method^42^ with spectroscopic property parameters combined with a configuration interaction scheme involving only single excitations. The electronic coupling calculation of the PE555 system is based on the transition dipole moment obtained by the quantum mechanical/molecular mechanics (QM/MM) method.^43,44^ The results obtained by this method are quite close to those from time-dependent density functional theory (TDDFT) calculations.^45^ When the electronic coupling is considered as coulomb interaction, calculated according to the point dipole approximation (PDA), some dipole moment results are derived from ZINDO/S-CIS calculations. The Hamiltonian of the system part of the PE555 complex, obtained based on the electronic coupling terms between pigments acquired by the PDA methods, is shown in Table 1.

**Table 1:** The site energies and the electronic coupling terms among chromophores in the PE555 complex (in cm^−1^),with the Hamiltonian obtained *via* the PDA approximation method

Site	1	2	3	4	5	6	7	8
PEB20A	18 688	9.094	10.812	35.594	−11.351	6.043	7.130	2.654
DBV50/61B	9.094	17 921	41.181	10.813	2.548	72.484	17.778	2.910
PEB82B	10.812	41.181	18 494	7.259	−6.452	17.686	3.390	2.268
PEB158B	35.594	10.813	7.259	18 881	2.887	3.145	−0.748	2.349
PEB20C	−11.351	2.548	−6.452	2.887	18 921	13.209	11.806	36.096
DBV50/61D	6.043	72.484	17.686	3.145	13.209	18 131	−38.772	24.991
PEB82D	7.130	17.778	3.390	−0.748	11.806	−38.772	18 284	7.119
PEB158D	2.654	2.910	2.268	2.349	36.096	24.991	7.119	18 808

Meanwhile, to further compare the electronic coupling terms between pigment molecules obtained by different methods, Ulrich *et al.*^41^ adopted the TrEsp method (where TrEsp stands for transition charges from electrostatic potential), which includes environmental effect in the polarizable continuum model (PCM).^46^ Based on this method, another exciton Hamiltonian of the PE555 complex is listed in Table 2. It should be noted that the TrEsp and PDA methods are theoretically related. When the distance between pigments is sufficiently large, the coulomb coupling calculated by TrEsp converges to the PDA result. However, for certain closely spaced pigment pairs in the PE555 complex, the accurate electronic coupling significantly deviates from the PDA prediction, highlighting the necessity of comparing the two methods.

**Table 2:** The Hamiltonian obtained by the TrEsp method, and the site energies of the PE555 complex and the electronic coupling between its chromophores (in cm^−1^)

Site	1	2	3	4	5	6	7	8
PEB20A	18 688	14.2	10.46	26.22	−10.54	−10.34	9.40	3.54
DBV50/61B	14.2	17 921	−40.69	13.75	−7.82	11.99	16.63	2.33
PEB82B	10.46	−40.69	18 494	6.98	−4.85	16.29	3.12	2.47
PEB158B	26.22	13.75	6.98	18 881	3.67	2.7	−0.048	2.33
PEB20C	−10.54	−7.82	−4.85	3.67	18 921	18.29	11.57	28.88
DBV50/61D	−10.34	11.99	16.29	2.7	18.29	18 131	−39	19.24
PEB82D	9.4	16.63	3.12	−0.048	11.57	−39	18 284	7
PEB158D	3.54	2.33	2.47	2.33	28.88	19.24	7	18 808

To systematically evaluate the influence of different electronic coupling schemes on exciton dynamics, a comparative analysis of the PDA, TrEsp, and TDC methods is necessary. All three approaches compute chromophore–chromophore couplings based on intermolecular coulomb interactions and rely on quantum chemical calculations to obtain electronic structure information, such as transition dipole moments or transition densities; however, they differ in their levels of approximation and computational cost. In the PDA framework, the transition density is approximated as *a* point dipole, leading to high computational efficiency and suitability for systems with relatively large intermolecular separations. The TrEsp method, by fitting transition charges to reproduce *ab initio* electrostatic potentials, provides a more realistic representation of the spatial charge distribution and achieves higher accuracy at short distances and when environmental polarization effects are included. The TDC approach^47^ directly evaluates the coulomb interaction by integrating transition densities on a three-dimensional grid, thereby fully accounting for molecular shape and electron density overlap; it offers the highest accuracy but at significantly greater computational expense. Overall, both accuracy and computational cost increase progressively from PDA to TrEsp and further to TDC. Considering the relatively open structural arrangement of the PE555 complex, PDA and TrEsp provide a favorable balance between accuracy and computational feasibility, making them suitable for exciton dynamics simulations of this system, whereas TDC remains impractical for systems of this size. Clarifying the theoretical distinctions among these methods facilitates a more consistent interpretation of the dynamical differences arising from different Hamiltonian constructions. The excitonic coupling matrices employed in this work were constructed based on the data reported in Table 2 of the work of Chandrasekaran *et al.*^41^ In that reference, the Hamiltonian matrix is given in units of meV, where the upper off-diagonal elements correspond to the TrEsp couplings and the lower off-diagonal elements to the PDA couplings. In the present study, these coupling values were first converted from meV to cm^−1^. Furthermore, since the reported couplings^41^ had been multiplied by an environmental screening factor *f* = 0.69, we divided the tabulated values by 0.69 to recover the unscreened electronic coupling strengths. No additional QM/MM simulations or trajectory averaging were performed in this work. The coupling matrices used in Tables 1 and 2 were obtained following this unit conversion and rescaling procedure.

Harrop *et al.*^21^ determined the 1.8 Å high-resolution structure of PE555 through X-ray crystallography, clearly revealing its “open structure” characteristics. The excitonic coupling values calculated in this study using the PDA/TrEsp methods (Tables 1 and 2) can be correlated with the actual intermolecular distances in this structure (*e.g.*, the distance between PEB20A and PEB158B is approximately 1.2 nm). As shown in Table 3, we present the distances between all chromophore pairs, thereby validating that the larger and more broadly distributed coupling values obtained by the PDA method—attributed to the open structure—are consistent with the distance trends observed in the experimental structure.^21^

**Table 3:** Intermolecular distance of chromophores (in Å)

Site	1	2	3	4	5	6	7	8
PEB20A	0	28.73	33.22	19.35	28.59	29.3	18.39	39.6
DBV50/61B	28.73	0	22.15	22.88	28.73	19.91	33.62	30.81
PEB82B	33.22	22.15	0	35.4	18.48	25.14	43.32	30.56
PEB158B	19.35	22.88	35.4	0	39.64	31.51	30	47.65
PEB20C	28.59	28.73	18.48	39.64	0	29.72	33.39	19.64
DBV50/61D	29.3	19.91	25.14	31.51	29.72	0	21.98	23.07
PEB82D	18.39	33.62	43.32	30	33.39	21.98	0	35.41
PEB158D	39.6	30.81	30.56	47.65	19.64	23.07	35.41	0

In describing the dynamics of excitation energy transfer (EET) in a photosynthetic complex containing N pigments, each pigment is simulated as a two-level system to characterize its S0 → S1 transition (that is, the *Q*_*y*_ transition of bacteriochlorophyll). The total Hamiltonian of the EET dynamics is composed of three parts.

1 *H*_tot_ = *H*_s_ + *H*_*b*_ + *H*_sb_

Here, *H*_s_ and *H*_*b*_ represent the system Hamiltonian and bath Hamiltonian, respectively, while *H*_sb_ is the interaction Hamiltonian between the system and the bath. The Hamiltonian of the electronic system can be expressed as:2
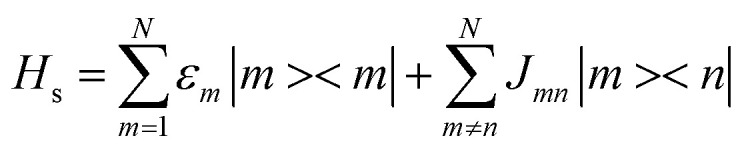
where, *ε*_*m*_, *J*_*mn*_, and *N* correspond to the electronic excitation energy of site m, the electronic coupling between site *m* and site *n*, and the total number of pigments, respectively. A single chromophore molecule is coupled to the protein environment, whose vibrational modes are regarded as a set of independent harmonic oscillators, with the corresponding bath Hamiltonian expressed as, 
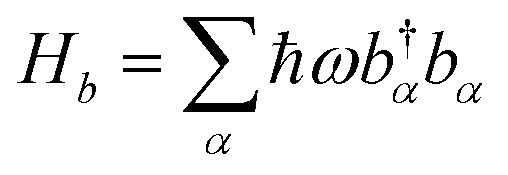
, here, *b*^†^_*α*_ and *b*_*α*_ are the creation operator and annihilation operator of the αth mode respectively. The interaction term between the system and the bath is noted as *H*_sb_, which contains both system quantities and environmental quantities. The interaction *H*_sb_ can be expressed in a bilinear form as: *H*_sb_ = ∑*m*|*m* > <*m*|*B*_*n*_, where *B*_*n*_ is the environmental operator.

The bath correlation function *C*(*t*) employed in our study belongs to the Drude–Lorentz type. According to the fluctuation-dissipation theorem, this function is connected to the environmental spectral density *J*(*ω*) as detailed below:^48^3
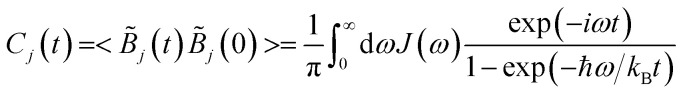


The spectral density of the system-bath coupling is defined as:
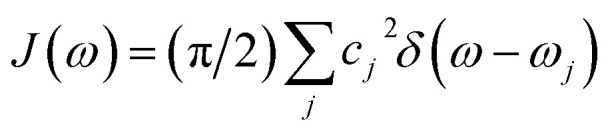
.^49^ This function determines all environmental correlation properties associated with the PE555 complex.

In this work, we employ the dissipation equation of motion (DEOM) method to investigate the excitation dynamics of the PE555 complex. The DEOM theory was recently proposed by Yan *et al.*^50–55^ as a nonperturbative approach for open quantum systems. It further extends the hierarchical equations of motion framework to describe the dynamical processes of a system coupled to its environment. This new theoretical framework not only encompasses the HEOM method, but also incorporates the environmental information directly interacting with the system, while defining all relevant dynamical quantities in terms of dissipative configurations and endowing them with clear physical meaning. In addition, it introduces a statistical quasiparticle concept for characterizing the environment, referred to as the dissipation. From this perspective, the DEOM method can be regarded as a second-quantization theory for open quantum systems, as it not only provides the equations governing dynamical evolution, but also establishes the corresponding statistical quasiparticle picture and a novel dissipative algebraic structure. Therefore, DEOM theory is considered to be an accurate and powerful theoretical tool for investigating the dynamics of open quantum systems in complex environments.

In the DEOM framework, the total Hamiltonian of the exciton-bath system is expressed as *H*_tot_ = *H*_s_ + *H*_*b*_ + *H*_sb_. Here, *H*_s_ is the exciton Hamiltonian of the PE555 complex, which contains the site excitation energies and the electronic coupling elements between different pigments. In the present work, two different forms of *H*_s_, obtained from the PDA and TrEsp methods, are used to examine the influence of the exciton Hamiltonian on the energy-transfer dynamics. The Hamiltonian acts on the reduced system and all dissipation density operators through the Liouvillian operator. Therefore, the coherent part of the DEOM propagation is given by −*i*[*H*(*t*), *ρ*_*T*_(*t*)], which directly governs the coherent exciton evolution, population redistribution, and quantum coherence between different chromophores. The bath-induced terms in the hierarchy account for the fluctuation, dissipation, and memory effects caused by the protein environment. In our simulations, the same bath spectral density parameters are used for the PDA and TrEsp Hamiltonians, so the observed differences in exciton dynamics originate from the different electronic coupling structures contained in *H*_s_.

This theory encompasses not only the dynamical rules for evolution variables but also reveals underlying statistical quasiparticle principles and novel dissipative algebraic structures. The dynamic variables of DEOM are called dissipative density operators, and their expressions are as follows:^56^4
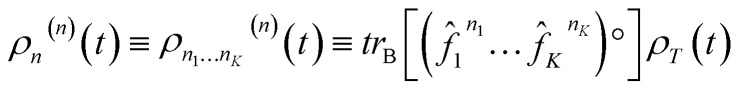


The dissipation inside (…)° irreducible and satisfy the bosonic commutator relation (*f̂*_*k*_…*f̂*_*k*^′^_)° = (*f̂*_*k*^′^_…*f̂*_*k*_)°. In eqn (4), *n* = *n*_1_ + … + *n*_*k*_, where *n*_*k*_ represents the number of a specific dissipation and *n*_*k*_ ≥ 0, with *n* being equivalent to *n* ≡ {*n*_1_…*n*_*K*_}, these parameters determine the composite structure of *n* dissipations, *ρ*_*n*_^(*n*)^, in eqn (4), while (*n* ± 1)-dissipations are respectively represented by 
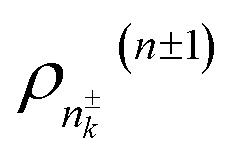
. The DEOM formalism is consequently expressed as:

5 *ρ̇*_*n*_^(*n*)^ = −[*iL*(*t*) + *γ*_*n*_^(*n*)^]*ρ*_*n*_^(*n*)^ + {*ρ*_*n*^−^_^(*n*−1)^} + {*ρ*_*n*^+^_^(*n*+1)^}

The formalism of this equation is derived by applying the Liouville equation *ρ̇*_*T*_(*t*) = −*i*[*H*(*t*), *ρ*_*T*_(*t*)], this density operator encompasses the total density operator of eqn (4). The method can provide accurate numerical results and verify the long-lived quantum coherent dynamics—this dynamic phenomenon has been observed in the PE555 complex under low temperature and ambient temperature environments, respectively. In addition, this method is reliable over a wide range of system-bath coupling strength and can yield reasonable numerical results.

For the spectral density calculations of the PE555 complex, researchers^21,41^ obtained the following spectral density parameters by numerically fitting experimental data: reorganization energy *λ* = 24.14 cm^−1^ and bath cutoff frequency *γ* = 56.82 cm^−1^. Based on this, we employed these parameters and the DEOM method to perform simulation calculations on the exciton dynamics process of the PE555 complex.

## Results and discussion

**Fig. 2:**
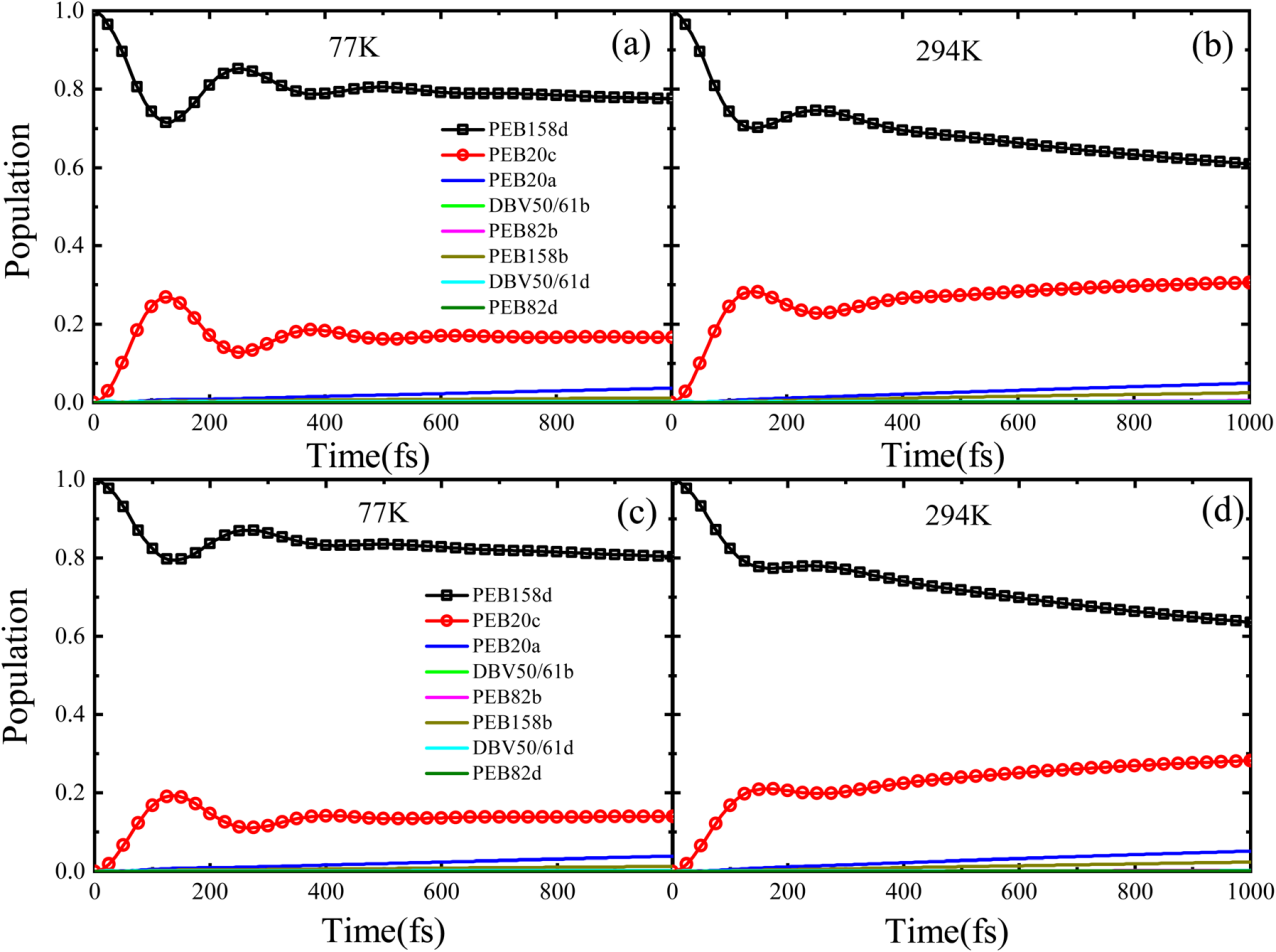
Population dynamics of the eight-pigment model with initial excitation of the PEB158D chromophore at temperatures of 77 K and 294 K, respectively. The results in panels (a) and (b) were obtained using the Hamiltonian derived *via* the PDA approximation method, while the results in panels (c) and (d) were obtained using the Hamiltonian derived by the TrEsp method.

First, we use the system Hamiltonian obtained by the PDA method using transition dipole moments (as shown in Table 1 above), and use this Hamiltonian to perform excitation dynamics calculations on the PE555 complex. We present the calculation results when the initial excitation is on the PEB158D chromophore, as shown in Fig. 2. The left side of the figure displays the calculation results at a low temperature of 77 K, and the right side shows the results at room temperature of 294 K. In the structure of the PE555 light harvesting system, the PEB158D chromophore molecule is located at the edge of the entire molecular structure, making it easy to receive light energy and get excited first. This structural position characteristic enables it to further promote the transfer of excitation energy to other chromophore molecules after relaxation within the PE555 complex. We use the Debye spectrum to describe the spectral density of the environment. Here, the coupling term between the system and the environment is represented by the reorganization energy *λ*, and the cutoff frequency is characterized by *γ*. Based on theoretical research and experimental results,^21,41^ we performed numerical calculations using the reorganization energy *λ* = 24.14 cm^−1^ and the cutoff frequency *γ* = 56.82 cm^−1^. When the PEB158D chromophore molecule is in the initial excited state, as shown in Fig. 2a, under the low temperature of 77 K, a quantum coherence phenomenon lasting about 400 femtoseconds can be observed between the PEB158D and PEB20C chromophore molecules. This is due to the relatively large coupling energy (36.1 cm^−1^) between these two chromophore molecules. Fig. 2b shows that the quantum coherence time remains at around 300 femtoseconds at a room temperature of 294 K. The calculation results indicate that there exists a quantum coherence phenomenon of approximately several hundred femtoseconds between the PEB158D and PEB20C chromophore molecules under both low temperature and room temperature conditions.

As previously mentioned, the electronic coupling terms of the PE555 complex can be obtained by two different methods. To compare the electronic coupling terms derived from these two methods, we constructed a Hamiltonian using the electronic coupling terms obtained by another method, namely the TrEsp method (as shown in Table 2 above). We performed exciton dynamics calculations based on this Hamiltonian. Using the DEOM method, we conducted calculations on the excitation dynamics process of the PE555 complex over a time scale of several tens of picoseconds under both low temperature and room temperature conditions, with the results presented in Fig. 2c and d. For the convenience of comparison, at a low temperature of 77 K, with PEB158D as the initially excited molecule, it can be observed from Fig. 2c that a period of coherent oscillation exists between PEB158D and PEB20C molecules. A coherent oscillation lasting roughly 300 femtoseconds occurs between the PEB158D and PEB20C molecules. Within 1000 femtoseconds, the energy initially flows to the PEB20A molecule. At room temperature, as can be seen from Fig. 2d, a certain degree of coherence still exists, the coherent oscillation between these two molecules endures for around 200 femtoseconds. However, a comparison with the previous results reveals that, regardless of low or room temperature, the results of exciton dynamics calculations on the PE555 complex using the exciton Hamiltonian approximated by the PDA method exhibit quantum coherence with a longer lifetime. In contrast, the calculation results using the exciton Hamiltonian obtained by the TrEsp method show that the quantum coherence lifetime between PEB158D and PEB20C molecules is relatively short, and the amplitude of coherent oscillation is also smaller. This may be due to the open structure of PE555 complex, which leads to differences in results obtained by different structural calculation methods; that is, the molecular structure of the PE555 complex has a certain influence on its excitation dynamics process.

**Fig. 3:**
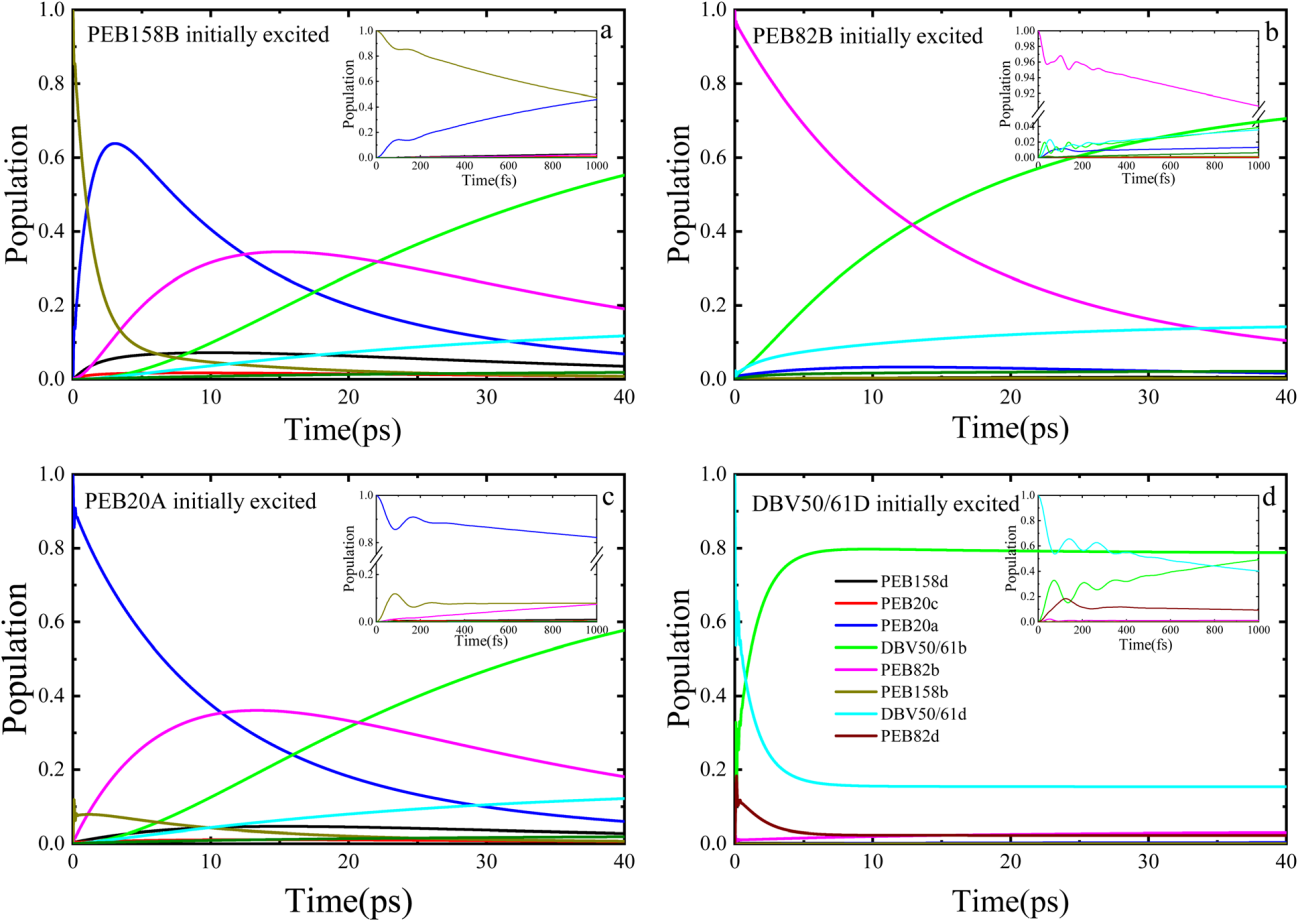
The population dynamics of the PE555 complex at *T* = 77 K simulated with initial excitation localized on the PEB158B, PEB82B, PEB20A, and DBV50/61D chromophores, respectively, by the PDA approximation method.

Fig. 3 illustrates the dynamic process of the population of the PE555 complex varying with time when four different chromophore molecules are excited at 77 K by the PDA approximation method. We performed the 40 picoseconds excitation dynamics calculation on the PE555 complex. When the PEB158B chromophore serves as the initially excited molecule, PEB20A responds rapidly. As depicted in Fig. 3a, within the first 100 femtoseconds, quantum coherent oscillations emerge between PEB158B and PEB20A molecules, after which the population of PEB158B chromophore molecules decays to zero within 25 picoseconds. Throughout the entire excitation dynamics process, the excitation energy of the excited PEB158B chromophore is transferred to the surrounding chromophores, with the energy ultimately flowing to the DBV50/61B molecule. When the PEB82B chromophore molecule is the initially excited one, as shown in Fig. 3b, the calculation results indicate that the amplitude of coherent oscillations is small and the coherence time is short. Around 12 picoseconds, a large quantity of excitation energy flows to the DBV50/61B molecule.

When the initially excited species are PEB20A or DBV50/61D chromophores, it can be observed that a certain population oscillation process exists in both scenarios, with the duration and amplitude of the oscillations being small. When the initial excitation is on the PEB20A molecule, as illustrated in Fig. 3c, the excitation energy first transfers to the PEB158B molecule, then gradually to PEB82B, and over time, the energy finally accumulates on the acceptor molecule DBV50/61B. Coherent oscillations exist between the PEB20A and PEB158B molecules, albeit with small amplitude and short duration. The energy first transfers to PEB158B, then gradually to PEB82B, and finally accumulates on DBV50/61B within a relatively short time (approximately 30 ps). The presence of short-lived coherence aids the initial energy redistribution. When the DBV50/61D chromophore molecule is initially excited, a quantum coherence phenomenon persists for a certain time. As shown in Fig. 3d, within a short period, the energy flows to the DBV50/61B molecule, and at approximately 5 picoseconds, the entire system attains the final steady state.

**Fig. 4:**
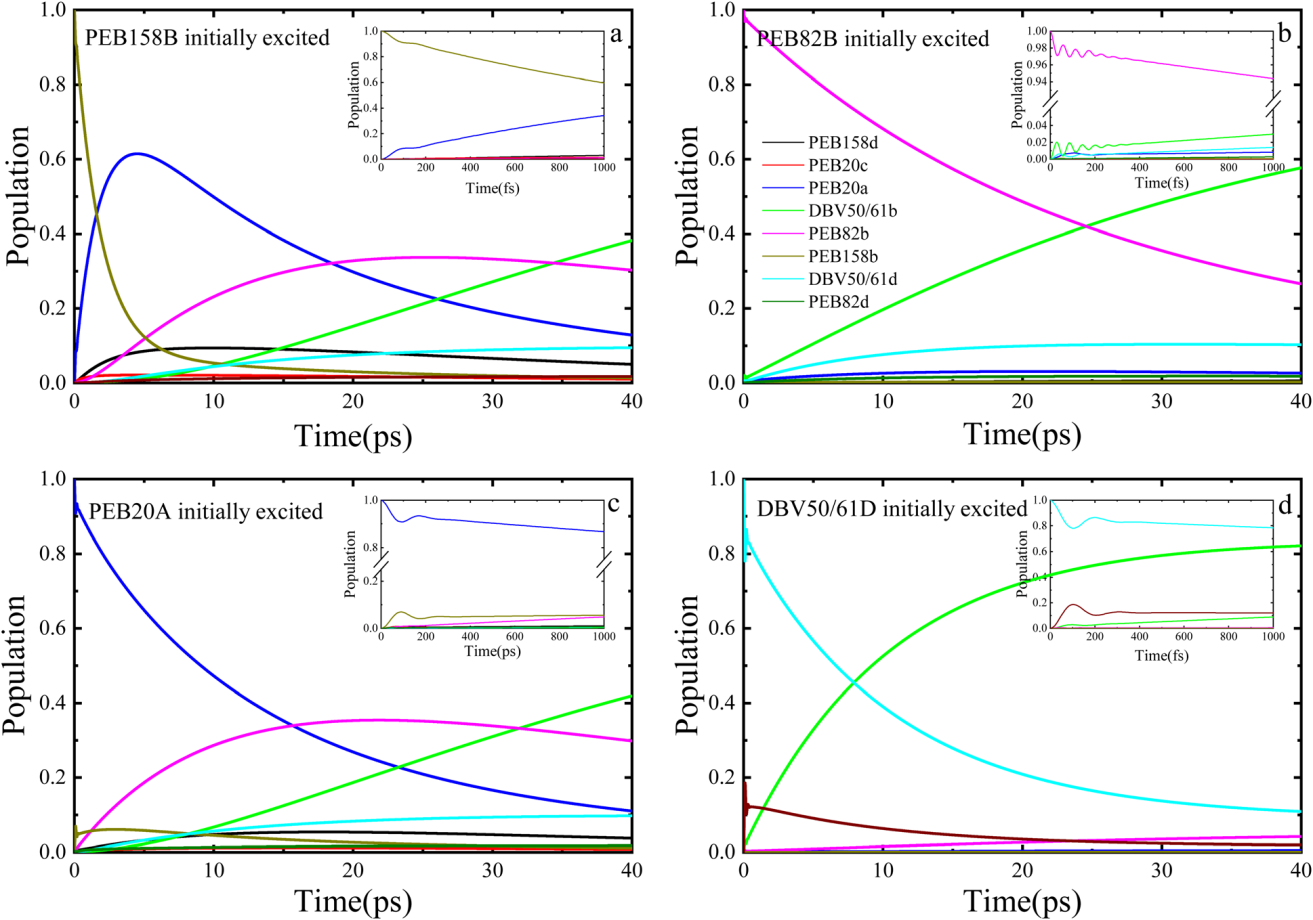
Population dynamics simulations of the PE555 complex at 77 K under conditions of initial excitation on chromophores PEB158B, PEB82B, PEB20A, and DBV50/61D, respectively, by the TrEsp method.

In addition, we also present the calculation results of the entire excitation dynamics process of the PE555 system when the PEB158B chromophore molecule, PEB82B molecule, PEB20A molecule, and DBV50/61D chromophore molecule are initially excited, respectively, by the TrEsp method. Under the low temperature condition of 77 K, we performed exciton dynamics calculations on the PE555 complex over several tens of picoseconds using the DEOM theoretical method. When the PEB158B molecule is initially excited, the calculation results are shown in Fig. 4a: after the PEB158B molecule is excited, it will generate short-term coherent oscillations with the PEB20A molecule, and then a large amount of excitation energy flows to the PEB82B molecule. At approximately 35 picoseconds, the energy starts to flow to the DBV50/61B molecule. As shown in Fig. 4b, when the PEB82B chromophore molecule is initially excited, most of the energy directly flows to the DBV50/61B molecule; with the evolution of time, part of the excitation energy flows to the DBV50/61D molecule, but the vast majority of the energy still flows to the DBV50/61B molecule. At around 26 picoseconds, the population of the excited PEB82B molecule is less than that of the DBV50/61B molecule, which indicates that from this point onward, the excitation energy begins to gradually accumulate in the DBV50/61B molecule. When the PEB20A molecule is initially excited (as shown in Fig. 4c), there is a coherent phenomenon between the PEB20A and PEB158B molecules, but the coherence amplitude is relatively small; subsequently, the population on the PEB82B molecule gradually increases. Between 15 picoseconds and 30 picoseconds, the excitation energy is mainly concentrated on the PEB82B molecule, and after 32 picoseconds, the excitation energy gradually accumulates entirely in the DBV50/61B molecule. Meanwhile, we also initially excited the DBV50/61D chromophore molecule, and the calculation results are shown in Fig. 4d. At approximately 8 picoseconds, the population of the DBV50/61B molecule begins to gradually increase, and finally, at 30 picoseconds, most of the excitation energy flows to the DBV50/61B molecule.

The researcher^21^ inferred from low-temperature absorption spectroscopy that the characteristic excitation energy transfer time in PE555 is approximately 10–50 ps; the population dynamics calculated using DEOM in this study (Fig. 3 and 4) demonstrate that the transfer from the initial excited state to DBV50/61B (the final acceptor) reaches steady state in about 25–40 ps (for instance, when PEB158B is initially excited, energy flows to the DBV50/61B molecule after 35 ps), theoretically validating the consistency between the theoretical dynamics and experimental trends.

**Fig. 5:**
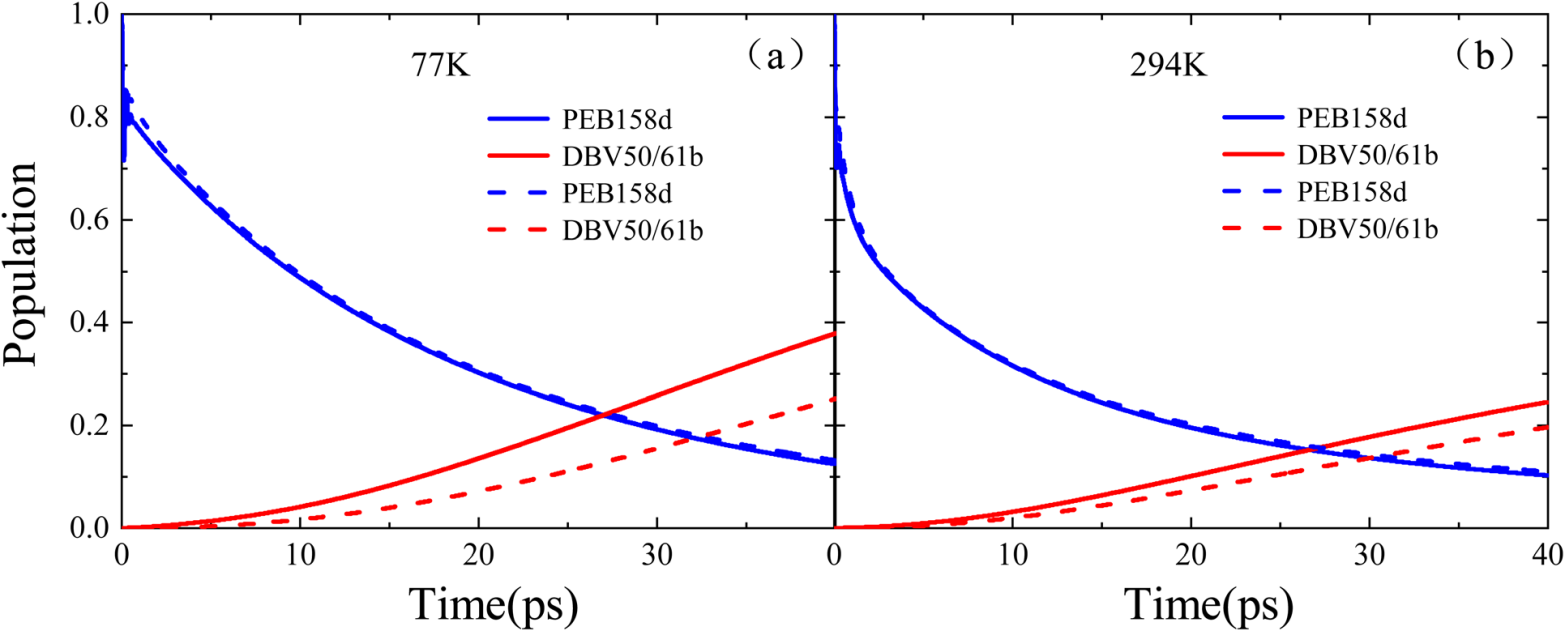
A comparison of the population evolution over time for the PE555 complex under two different Hamiltonians: the solid line represents results calculated using the Hamiltonian derived from the PDA approximation method, while the dashed line shows results from calculations with the Hamiltonian obtained by the TrEsp method.

From the previous discussion, it can be seen that in the process of excitation energy transfer in the PE555 complex, the final molecule to which the energy flows the DBV50/61B molecule. This paper primarily focuses on the influence of the system Hamiltonian, obtained from electronic coupling terms calculated using two different methods, on the excitation energy transfer process. Furthermore, we calculated the exciton dynamics of this complex using these two Hamiltonians, respectively, and discussed the amount of population finally flowing to the acceptor molecule and the speed of excitation energy flowing to the DBV50/61B molecule. At 77 K, the PEB158D pigment molecule was first excited, and the calculation results are shown in Fig. 5a. The solid line represents the calculation result using the Hamiltonian in Table 1 (*i.e.*, the exciton Hamiltonian obtained by the PDA approximation method), and the dashed line represents the calculation result using the data in Table 2 (*i.e.*, the Hamiltonian obtained by the TrEsp method). It shows that when using the data in Table 1 for calculation, the population on the DBV50/61B molecule starts to exceed that of the donor molecule PEB158D pigment molecule at about 27 picoseconds. Then, a large amount of excitation energy flows to the acceptor molecule. The dashed line shows that at about 32 picoseconds, the population on the DBV50/61B molecule is higher than that of the donor molecule. In terms of the time accumulation rate, it is found that when using the Hamiltonian with data from Table 1, the excitation energy of the PE555 complex flows to the acceptor molecule faster. Meanwhile, it can be seen that at 40 picoseconds, the final population flowing to the DBV50/61B molecule is about 0.4, while when using the Hamiltonian in Table 2, the final population accumulated on the DBV50/61B molecule is about 0.25.

Similarly, at 294 K, we calculated the excitation dynamics of PE555 complex using the same method, and the results are shown in Fig. 5b. From the 40 picosecond simulation calculation, it can be seen that whether using the Hamiltonian in Table 1 or the data in Table 2, the final population on the acceptor molecule DBV50/61B is much lower than that accumulated at low temperature. This is because the excitation energy transfer process is affected by temperature at room temperature; the higher the temperature, the more complex the environmental fluctuations, which in turn affect the excitation dynamics process. However, even at room temperature of 294 K, we can still observe the result of the solid line, that is, when using the Hamiltonian data in Table 1, the population of the PE555 complex finally flowing to the acceptor molecule is more than that when using the data in Table 2 (as shown by the dashed line). Through the comparison of the results, it is found that the Hamiltonian obtained by the PDA approximation method (*i.e.*, the Hamiltonian in Table 1) enables the PE555 complex to transfer excitation energy more quickly and efficiently. We analyze that the reason is that the coupling distribution between various chromophores obtained by the PDA approximation is wider than that obtained by the TrEsp method, and the open structure of the PE555 complex is also an important factor.

In a word, we employed the DEOM theoretical method to calculate the excitation energy transfer process in the PE555 light harvesting complex, based on exciton Hamiltonians obtained under various conditions. We adopted the Drude spectral density form and performed numerical calculations in both room temperature and low temperature environments. The longer relaxation times observed in this work arise from the use of a Debye-type spectral density that does not explicitly include high-frequency vibrational modes. This modeling choice naturally leads to slower population redistribution compared with atomistic treatments. The difference reflects distinct system-bath assumptions rather than any limitation of the theoretical framework. The simulation time of 40 ps was adopted to ensure convergence of the steady-state population. The results show that regardless of the Hamiltonian used under different conditions, there exist quantum coherent oscillation signals with a certain lifetime at low temperatures; even at room temperature, there is a quantum coherence phenomenon lasting about 300 femtoseconds between the PEB158D and PEB20C chromophores. Meanwhile, a comparison of the calculation results using Hamiltonians under two different conditions reveals that when the exciton Hamiltonian obtained by the point dipole approximation (PDA) method is used for excitation energy calculation, the quantum coherence time between PEB158D and PEB20C molecules is longer, and the coherence amplitude is larger, whether at low temperature or room temperature. In addition, we also excited other chromophore molecules (such as PEB158B chromophore, PEB82B molecule, *etc.*) and used the DEOM method to perform 40 picosecond exciton dynamics calculations on the PE555 complex. The results similarly indicate that when using the Hamiltonian in Table 1 (*i.e.*, the exciton Hamiltonian obtained by the PDA approximation method), the population of the PE555 complex finally flowing to the acceptor molecule is larger. This result suggests that the open structure of the PE555 complex leads to weak intermolecular coupling, while the electronic coupling terms obtained by PDA coupling have larger values and a wider distribution. This enables the excitation dynamics process of the PE555 complex under such conditions to transfer more excitation energy to the acceptor molecule more quickly, which is then transmitted to the reaction center.

Additionally, we further analyzed the population number of the final acceptor molecule DBV50/61B and the rate at which the excitation energy flows to this molecule when calculating the excitation dynamics of the PE555 complex using the exciton Hamiltonians obtained by two different methods under low temperature and room temperature conditions. The calculation results show that at a low temperature of 77 K, when using the exciton Hamiltonian obtained by the PDA method, the population on the DBV50/61B molecule exceeds that on the donor molecule PEB158D at around 27 picoseconds, and reaches approximately 0.4 at 40 picoseconds. In contrast, when using the Hamiltonian obtained by the TrEsp method, it takes 32 picoseconds for the population on the DBV50/61B molecule to surpass that of the donor molecule, and the accumulated population on this molecule is only about 0.25 at 40 picoseconds. Through the comparison of the above results, it can be seen that the exciton energy transfer in the PE555 complex is faster and more efficient when calculated using the Hamiltonian obtained by the PDA approximation method. This is because the exciton coupling terms obtained by the PDA approximation are larger in value and wider in distribution compared to those obtained by the TrEsp method. Therefore, when using the exciton Hamiltonian obtained by the PDA approximation method for calculations, the PE555 complex can transfer a large amount of excitation energy to the final acceptor molecule more rapidly. In the present study, the faster population redistribution obtained with the PDA coupling primarily arises from its larger numerical magnitude within the current Hamiltonian, which enhances the effective electronic interactions under otherwise identical dynamical conditions. This difference reflects the quantitative characteristics of the coupling models rather than any intrinsic physical “preference” of the biological system for a particular coupling scheme. The purpose of our comparison is to elucidate how different coupling approaches influence exciton dynamics within the same theoretical framework, rather than to suggest that one model is physically superior to the other.

To further elucidate the underlying physical implications, it is important to note that exciton Hamiltonians constructed under different coupling schemes intrinsically correspond to distinct delocalization patterns and energy-level organizations. Variations in coupling strength modulate the degree of exciton delocalization and the distribution of excitation among pigments, thereby reshaping the effective energy landscape for excitation energy transfer (EET) and altering the dynamical balance between coherent evolution and environmental relaxation—an effect that is particularly evident within the DEOM framework. Consequently, differences in coherence lifetimes, population redistribution pathways, and the relative contributions of coherent and incoherent transport are not merely numerical discrepancies, but rather stem from qualitative distinctions in the physical picture of exciton transport. In other words, the manner in which the exciton Hamiltonian is constructed not only quantitatively affects dynamical predictions, but also fundamentally influences the physical interpretation of energy transfer mechanisms and coherence regulation.

## Conclusion

In summary, this study systematically compared the differences between exciton Hamiltonians constructed using the point dipole approximation (PDA) and the transition density cube (TrEsp) approach in describing the excitation energy transfer process of the PE555 light harvesting complex. By integrating theoretical calculations based on the dissipation equation of motion (DEOM) method, we not only confirmed the presence and persistence of quantum coherence effects in this system under both low temperature and room temperature conditions, and clarified their significant influence on exciton dynamics, but also elucidated the intrinsic mechanism by which the open structural configuration of PE555 finely regulates energy transfer efficiency and rate through modulation of the strength and spatial distribution of electronic coupling terms. Specifically, owing to its structural characteristics, the coupling elements derived from the PDA method generally exhibit larger magnitudes and broader distributions, leading to noticeable differences in dynamical predictions.

The results demonstrate that the construction scheme of the exciton Hamiltonian can qualitatively influence the predicted coherence preservation behavior, population dynamics, and overall energy transfer efficiency in open quantum systems. Therefore, an accurate physical description of excitonic coupling is essential for establishing reliable theoretical models of light harvesting systems. This work not only deepens the molecular level understanding of the energy regulation mechanism in PE555, highlighting the synergistic interplay between quantum coherence and structural features, but also provides a systematic theoretical framework and methodological guidance for investigating energy transfer mechanisms in other natural photosynthetic systems and for the rational design of highly efficient artificial light harvesting materials.

## Author contributions

Xueyan Cui: methodology, investigation, writing – original draft, writing – review & editing Zemin Sheng: conceptualization, methodology, writing – original draft Wei Song: methodology, investigation, writing – original draft Dengke Zhao: methodology, investigation, writing – original draft Yijing Yan: conceptualization, writing – review & editing Jianhua Wei: conceptualization, writing – review & editing.

## Conflicts of interest

There are no conflicts to declare.

## Data Availability

The data that support the findings of this study are available from the corresponding authors upon reasonable request.
