# Correction: Theoretical study on the influence of different exciton Hamiltonians on the excitation dynamic process of the PE555 complex

**DOI:** 10.1039/d6ra90048h

**Published:** 2026-05-19

**Authors:** XueYan Cui, ZeMin Sheng, Wei Song, Dengke Zhao, YiJing Yan, JianHua Wei

**Affiliations:** a Department of Science, Henan Institute of Technology Xinxiang 453003 China cxy@hait.edu.cn; b School of Materials Science and Engineering, Henan Normal University Xinxiang 453007 China; c Department of Chemical Physics, Hefei National Laboratory for Physical Sciences at the Microscale, University of Science and Technology of China Hefei Anhui 230026 China; d Department of Physics, Beijing Key Laboratory of Optoelectronic Functional Materials and Micro-nano Devices, Renmin University of China Beijing 100872 China

## Abstract

Correction for ‘Theoretical study on the influence of different exciton Hamiltonians on the excitation dynamic process of the PE555 complex’ by XueYan Cui *et al.*, *`*, 2026, **16**, 23870–23883, https://doi.org/10.1039/D5RA09996J

The authors regret that the affiliation for one of the authors (Wei Song) was shown incorrectly in the original article. The corrected affiliations are as shown above.

The Royal Society of Chemistry apologises for these errors and any consequent inconvenience to authors and readers.

